# Supplementary material for: Behavioral screening of conserved RNA-binding proteins reveals CEY-1/YBX RNA-binding protein dysfunction leads to impairments in memory and cognition
Source: bioRxiv. 2024 Jan 6:2024.01.05.574402. Preprint. [Version 1] doi: 10.1101/2024.01.05.574402 (PMC10802296; doi:10.1101/2024.01.05.574402)
Supplement: Supplementary file 1 [file media-1.pdf]

Supplemental Figure 1

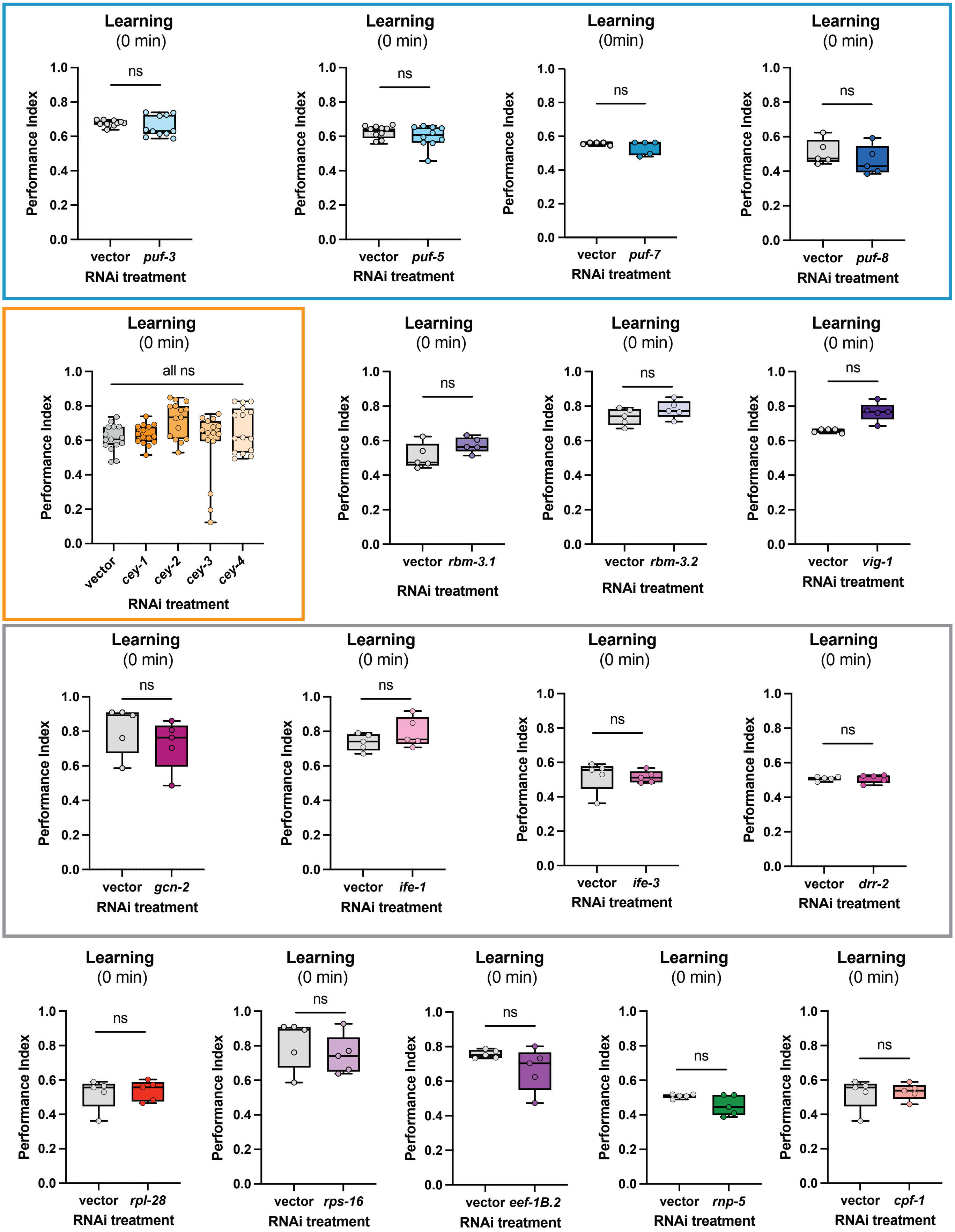

Supplemental Figure 2

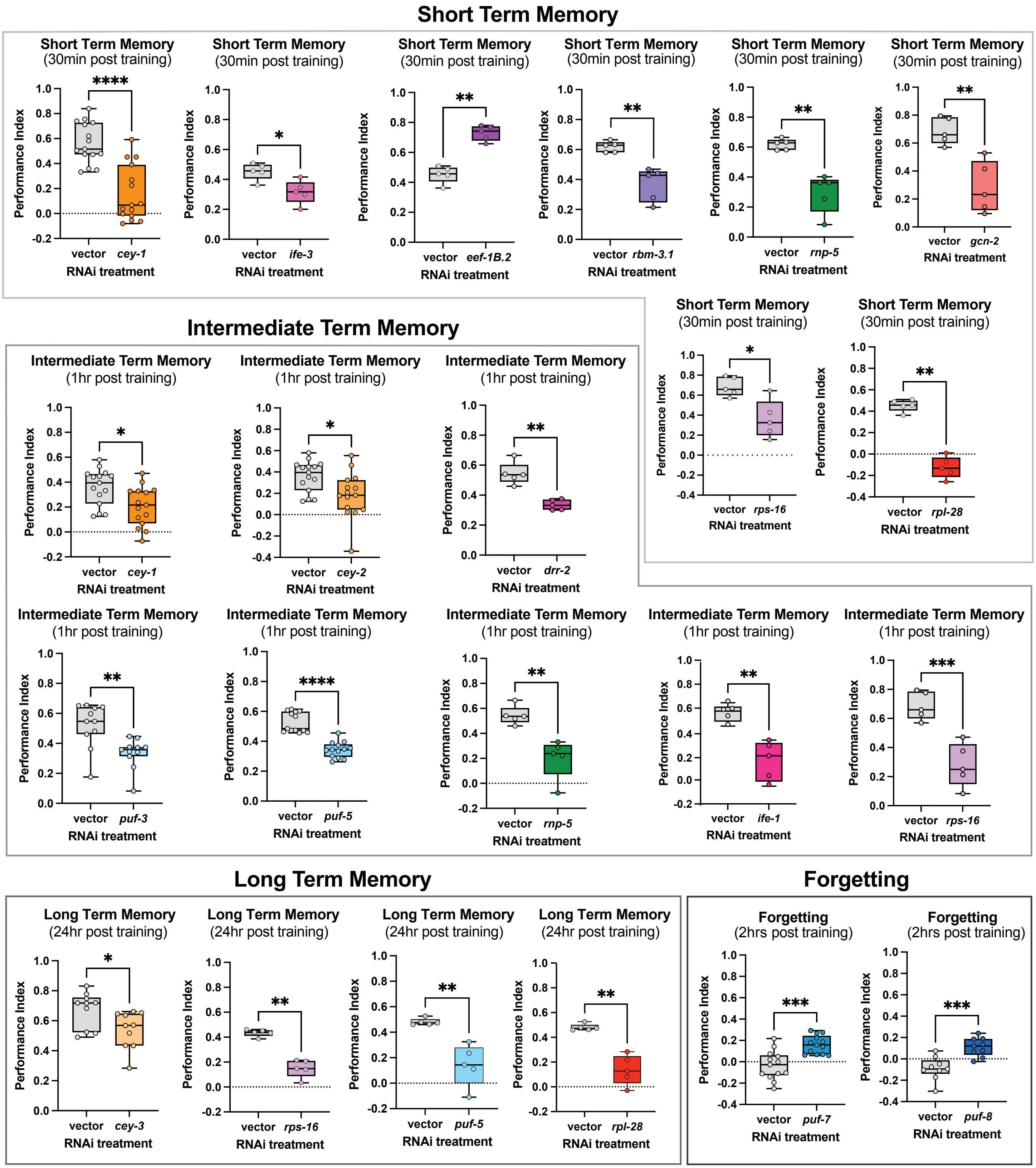

Supplemental Figure 3

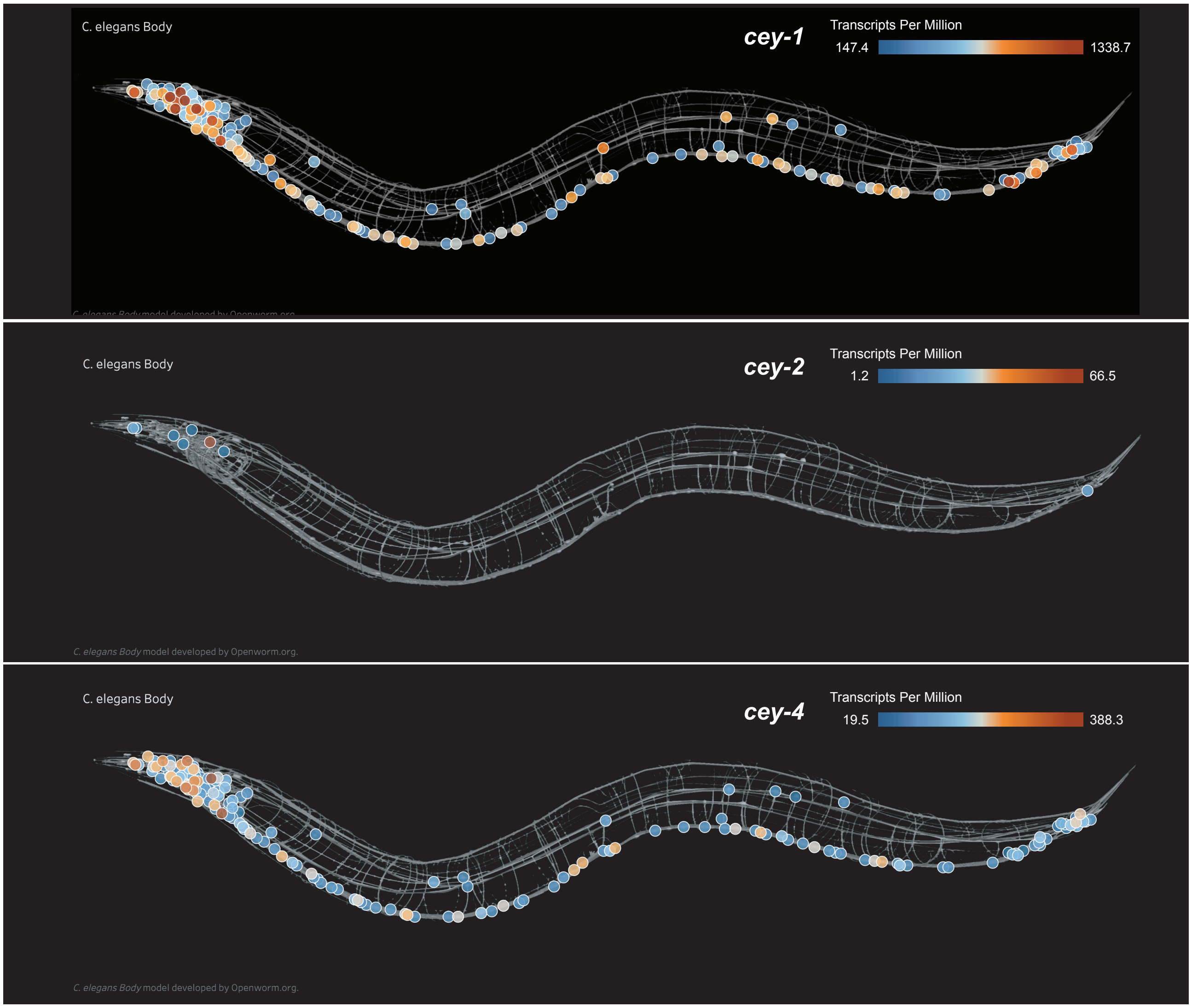

Supplemental Figure 4

A

Publication Dataset	Type of Sequencing	*cey-1* mRNA present?
Kaletsky et al. 2018	Neuron-specific RNA-seq in Day 1 adults	Yes
Yao et al. 2018	Integrated neuron-specific transcriptomics from over 2400 publications	Yes
Arey et al. 2019	Nervous system-wide presynaptic RNAseq in Day 1 adults	Yes
*Taylor et al. 2021**	Single-cell RNA-seq in neurons of L4 larvae	Yes (42.4%)
Wang et al. 2022	Neuron-specific Smart-seq2 in Day 1 and Day 8 adults	Yes

**Percentage of neurons transcript is detected in assuming 302 neurons with a stringency of 2*

B

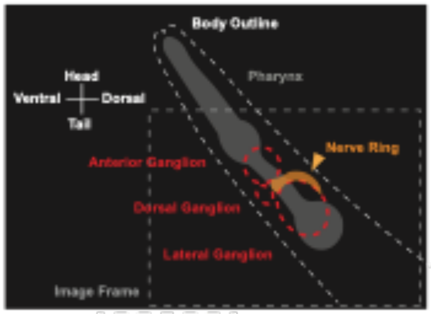

C

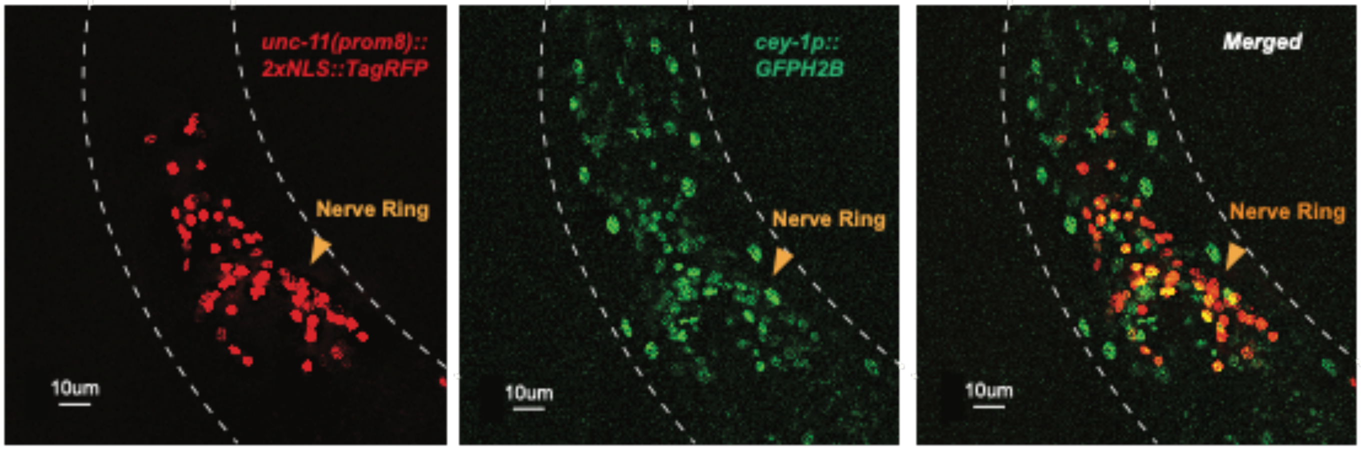

Supplemental Figure 5

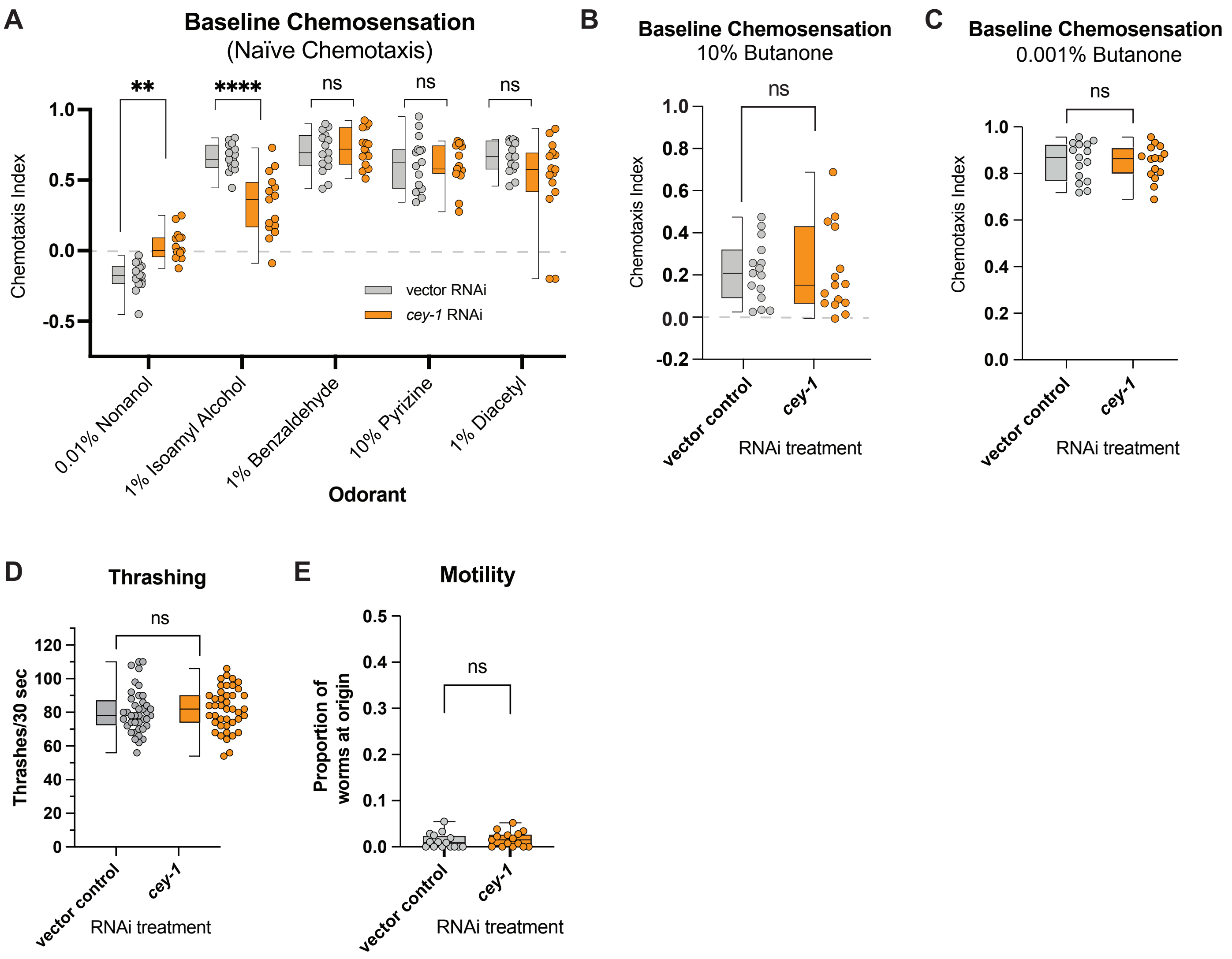

Supplemental Figure 6

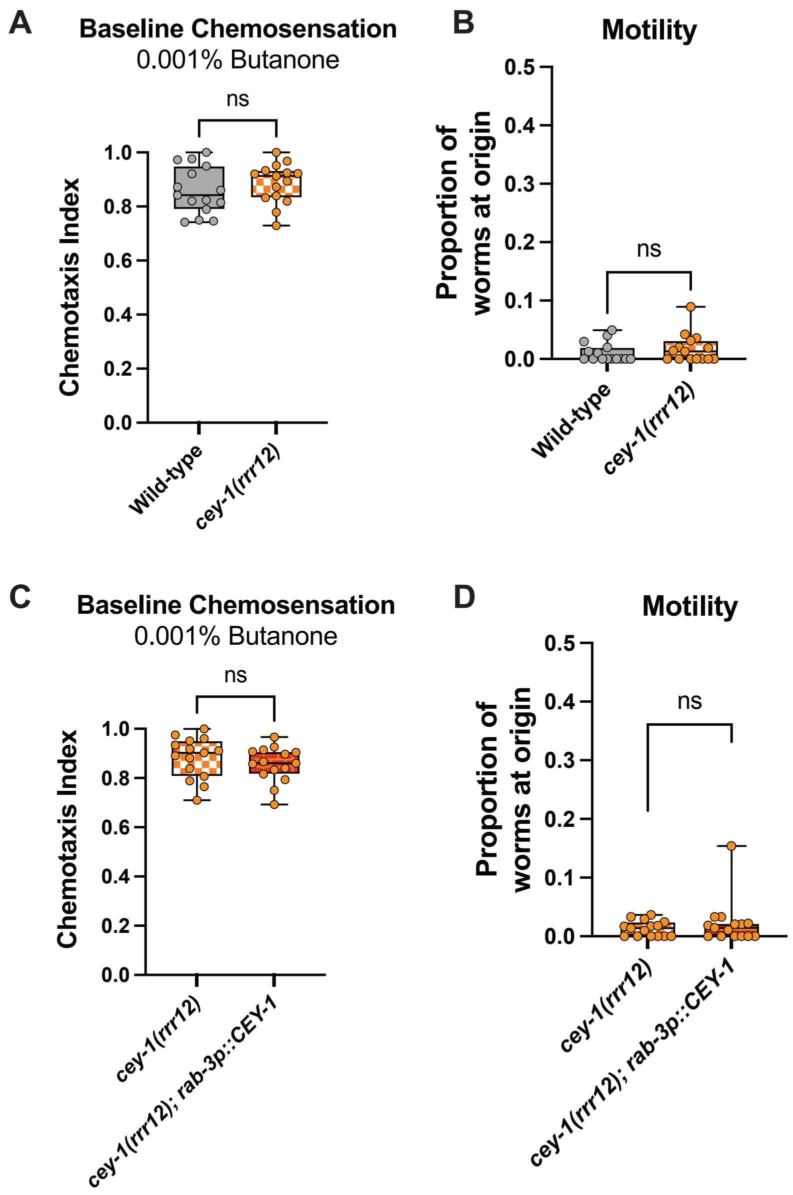

Supplemental Figure 7

CNV gains and losses affecting *YBX1* in DECIPHER v.11.22 (10/23)

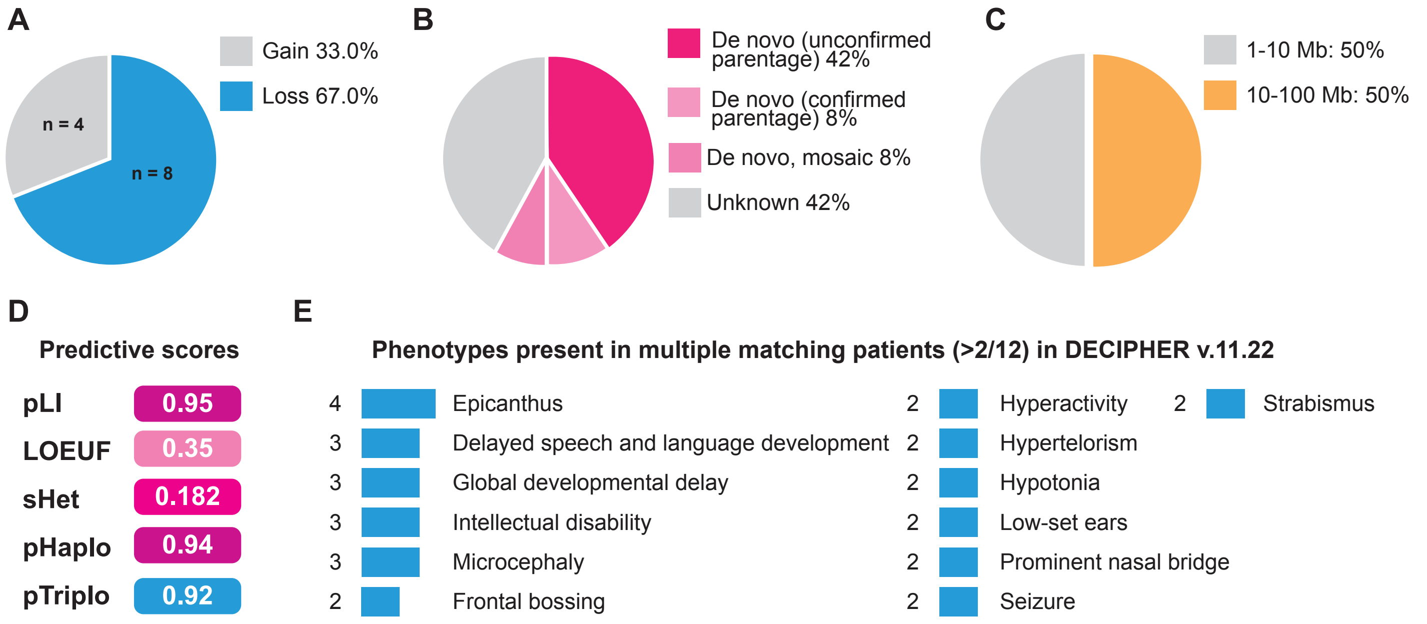

F

CNV losses affecting *YBX1* in DECIPHER v.11.22

Sex	Size	Inheritance	Genotype	Genome	Coordinates	Phenotype
46XX	24.59 Mb	Unknown	Heterozygous	GRCh38	Chr1:36366122-60952096	Hypoplasia of the corpus callosum; Intellectual disability
46XX	1.74 Mb	De novo	Heterozygous	GRCh38	Chr1:41803874-43541650	Intellectual disability; Microcephaly; Spastic diplegia
46XX	1.82 Mb	De novo	Heterozygous	GRCh38	Chr1:41352287-43169173	Delayed speech and language development; Global developmental delay; Microcephaly; Seizure; Thick lower lip vermilliion; Upslanted palpebral fissure
46XY	4.01 Mb	De novo	Heterozygous	GRCh38	Chr1:40660693-44668040	Primary microcephaly; Short stature
46XX	1.59 Mb	De novo	Heterozygous	GRCh38	Chr1:41649528-43242601	Ataxia; Atypical behavior; Global developmental delay; Seizure; Sleep disturbance
46XY	11.76 Mb	Unknown	Heterozygous	GRCh38	Chr1:34165306-45922308	Deep philtrum; Epicanthus; Hyperactivity; Pectus excavatum; Prominent nasal bridge; Short philtrum
46XX	11.76 Mb	Unknown	Heterozygous	GRCh38	Chr1:34165306-45922308	Epicanthus; Hyperactivity; Macrodontia; Narrow forehead; Prominent nasal bridge
46XY	3.90 Mb	De novo	Heterozygous	GRCh38	Chr1:38992486-42890360	Abnormal columella morphology; Brachycephaly; Caesarian section; Coarse hair; Depressed nasal ridge; Epicanthus; High anterior hairline; Hypoplastic philtrum; Hypotonia; Intellectual disability; Large earlobe; Low-set ears; Microcephaly; Micrognathia; Prominent ear helix; Short palpebral fissure; Strabismus; Talipes equinovarus; Wide nasal base

Supplemental Figure 8

CNV gains and losses affecting *YBX3* in DECIPHER v.11.22 (10/23)

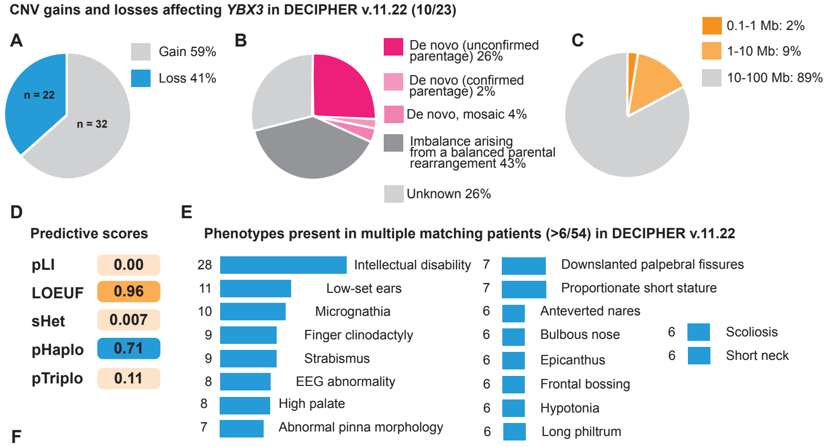

F

CNV losses affecting *YBX3* in DECIPHER v.11.22

Sex	Size	Inheritance	Genotype	Genome	Coordinates	Phenotype
Unknown	5.52 Mb	Unknown	Heterozygous	GRCh38	Chr12:8359134-14062991	Hypoplasia of the maxilla; Intellectual disability; Low anterior headline; Prominent supraorbital ridges; Short foot; Short palm; Short stature; Synophrys
46XY	3.25 Mb	De novo (unconfirmed parantage)	Heterozygous	GRCh38	Chr12:10330666-13580527	None listed
46XY	14.61 Mb	Imbalance arising from balanced parental rearrangement	Heterozygous	GRCh38	Chr12:45001-14655799	Arachnodactyly; Asymmetry of the thorax; Ataxia; Deeply set eye; Down-sloping features; Flexion contracture; Frontal bossing; Full cheeks; Intellectual disability; Kyphosis; Low posterior hairline; Low-set ears; Microtia; Muscle fiber atrophy; Pectus carinatum; Priminent metopic ridge; Proportionate short stature; Proximal placement of thumb; Round face; Scoliosis; Short neck; Single transverse palmar crease; Talipes equinovarus; Wide intermamillary distance
46XX	14.61 Mb	Imbalance arising from balanced parental rearrangement	Heterozygous	GRCh38	Chr12:45001-14655799	Abnormal pinna morphology; Abnormal thumb morphology; Arachnodactyly; Chorioretinitis; Deeply set eye; Edema of the dorsum of feet; EEG abnormality; Epicanthus; Hemiplegia; High palate; Hyperconvex nail; Hypotonia; Incoordination; Intellectual disability; Joint laxity; Kyphosis; Long hallux; Low-set ears; Narrow forehead; Nystagmus; Scoliosis; Short sternum; Strabismus; Talipes equinovalgus; Tooth malposition
46XY	14.61 Mb	Imbalance arising from balanced parental rearrangement	Heterozygous	GRCh38	Chr12:45001-14655799	Abnormal pinna morphology; Arachnodactyly; Asymmetry of the thorax; Bulbous nose; Cryptochidism; Deeply set eye; Down-sloping shoulders; Elbow flexion contracture; Epicanthus; Facial asymmetry; Full cheeks; Intellectual disability; Kyphosis; Limited shoulder movement; Low posterior hairline; Low-set ears; Microcephaly; Micropenis; Muscle fiber atrophy; Pectus carinatum; Prominent metopic ridge; Proportionate short stature; Proximal placement of thumb; Ptosis; Round face; Scoliosis; Short neck; Single transverse palmar crease; Small scrotum; Wide intermamillary distance
46XX	14.61 Mb	Imbalance arising from balanced parental rearrangement	Heterozygous	GRCh38	Chr12:45001-14655799	Abnormal pinna morphology; Atrial septal defect; Bulbous nose; Downslanted palpebral fissues; Finger clinodactyly; Flexion contracture; Frontal bossing; High palate; Intellectual disability; Mandibular prognathia; Prominent glabella; Protruding ear; Strabismus; Talipes equinovarus; Ventricular septal defect
46XX	14.61 Mb	Imbalance arising from balanced parental rearrangement	Heterozygous	GRCh38	Chr12:45001-14655799	Abnormal pinna morphology; Atrial septal defect; Bulbous nose; Downslanted palpebral fissues; Finger clinodactyly; Flexion contracture; Frontal bossing; High palate; Intellectual disability; Mandibular prognathia; Prominent glabella; Protruding ear; Strabismus; Talipes equinovarus; Ventricular septal defect
46XX	14.61 Mb	Imbalance arising from balanced parental rearrangement	Heterozygous	GRCh38	Chr12:45001-14655799	Abnormal pinna morphology; Abnormality of the face; Atrial septal defect; Bulbous nose; Dental malocclusion; Downslanted palpebral fissures; Flexion contracture; Frontal bossing; High palate; Hyperextensibility of the finger joints; Intellectual disability; Lethargy; Low posterior hairline; Mandibular prognathia; Open mouth; Overlapping toe; Prominent glabella; Protruding ear; Recurrent infections; Rocker bottom foot; Scoliosis; Small hand; Strabismus; Tapered finger
46XY	14.61 Mb	Imbalance arising from balanced parental rearrangement	Heterozygous	GRCh38	Chr12:45001-14655799	Abnormal pinna morphology; Bulbous nose; Cleft palate; Congenital diaphragmatic hernia; Cryptorchidism; Downslanted palpebral fissures; Finger clinodactyly; Flexion contracture; Frontal bossing; High palate; Hydrocephalus; Intellectual disability; Mandibular prognathia; Non-midline cleft lip; Open mouth; Pectus excavatum; Prominent glabella; Protruding ear; Recurrent infections; Strabismus
46XY	14.61 Mb	Imbalance arising from balanced parental rearrangement	Heterozygous	GRCh38	Chr12:45001-14655799	Abnormality of prenatal development or birth; Anteverted nares; Arachnodactyly; Bifid uvula; Downslanted palpebral fissures; Enlarged naris; Epicanthus; Finger clinodactyly; High palate; Intellectual disability; Long hallux; Long philtrum; Macrotia; Microcephaly; Micrognathia; Proportionate short stature; Slender finger; Small for gestational age; Tooth malposition; Webbed neck; Wide intermamillary distance
46XX	21.11 Mb	De novo (unconfirmed parantage)	Heterozygous	GRCh38	Chr12:45001-21155799	Abnormal lens morphology; Anteverted nares; Choanal atresia; Delayed closure of the anterior fontanelle; Ectopic anus; Flexion contracture; Hemivertebrae; High palate; Hyperconvex nail; Hypoplasia of the corpus callosum; Hypotonia; Intellectual disability; Large for gestational age; Long philtrum; Macrocephaly at birth; Microcornea; Micrognathia; Midface retrusion; Narrow palate; Omphalocele; Overlapping toe; Proptosis; Scoliosis; Short neck; Single transverse palmar crease; Small nail; Ulnar deviation of finger; Vertebral segmentation defect; Webbed neck
46XY	14.61 Mb	De novo (unconfirmed parantage)	Heterozygous	GRCh38	Chr12:45001-14655799	Abnormality of the face; Delayed skeletal maturation; Intellectual disability; Low-set ears; Primary microcephaly; Prominent nose; Short stature; Small for gestational age
46XX	14.61 Mb	De novo (unconfirmed parantage)	Heterozygous	GRCh38	Chr12:45001-14655799	Intellectual disability; Micrognathia; Overlapping toe; Protruding tongue; Strabismus; Tooth malposition; Ventricular septal defect; Wide mouth; Wide nasal bridge
46XX	14.61 Mb	Imbalance arising from balanced parental rearrangement	Heterozygous	GRCh38	Chr12:45001-14655799	Aplasia cutis congenita of scalp; Cleft palate; Finger clinodactyly; Hypertelorism; Hypotonia; Micrognathia; Short palpebral fissure; Small for gestational age; Ventricular septal defect
46XY	14.61 Mb	Imbalance arising from balanced parental rearrangement	Heterozygous	GRCh38	Chr12:45001-14655799	Intellectual disability; Strabismus
46XY	14.61 Mb	Imbalance arising from balanced parental rearrangement	Heterozygous	GRCh38	Chr12:45001-14655799	Intellectual disability
46XY	14.61 Mb	Imbalance arising from balanced parental rearrangement	Heterozygous	GRCh38	Chr12:45001-14655799	EEG abnormality; Intellectual disability
46XY	14.61 Mb	Imbalance arising from balanced parental rearrangement	Heterozygous	GRCh38	Chr12:45001-14655799	EEG abnormality; Intellectual disability
46XY	14.61 Mb	Imbalance arising from balanced parental rearrangement	Heterozygous	GRCh38	Chr12:45001-14655799	Abnormal shoulder morphology; Barrel-shaped chest; Broad philtrum; Bulbous nose; Cryptorchidism; Deep philtrum; Dolichocephaly; Downslanted palpebral fissures; EEG abnormality; Everted lower lip vermilion; Facial asymmetry; Finger clinodactyly; Hypertelorism; Intellectual disability; Large earlobe; Large hands; Long neck; Low-set ears; Macrotia; Malar flattening; Micrognathia; Narrow nasal septum; Pes planus; Posteriorly rotated ears; Prominent antihelix; Prominent nose; Proportionate short stature; Protruding ear; Sandal gap; Short phalanx of finger; Short philtrum; Small scrotum; Talipes equinovalgus; Thick upper lip vermilion; Thin upper lip vermilion; Webbed neck; Wide mouth; Wide nasal base; Wide nasal bridge
46XY	14.61 Mb	Imbalance arising from balanced parental rearrangement	Heterozygous	GRCh38	Chr12:45001-14655799	EEG abnormality; Intellectual disability; Kyphosis
46XX	14.61 Mb	Imbalance arising from balanced parental rearrangement	Heterozygous	GRCh38	Chr12:45001-14655799	Aplasia of the phalanges of the hand; Cutaneous finger syndactyly; Macrotia; Malar flattening; Prematurely aged appearance; Short nose; Small nail; Wide nasal base
46XX	14.61 Mb	Imbalance arising from balanced parental rearrangement	Heterozygous	GRCh38	Chr12:45001-14655799	Abnormality of the dentition; Broad neck; Broad palm; Delayed speech and language development; Epicanthus; Finger clinodactyly; Intellectual disability; Malar flattening; Metatarsus adductus; Microcephaly; Microdontia; Recurrent infections; Sandal gap; Short neck; Small hand; Strabismus; Synophrys
46XX	3.88 Mb	De novo (unconfirmed parantage)	Heterozygous	GRCh38	Chr12:10342477-14226152	Deeply set eye; Fine hair; Frontal bossing; Hydrocephalus; Micrognathia; Postnatal macrocephaly; Premature birth; Slender build

Supplemental Figure 9

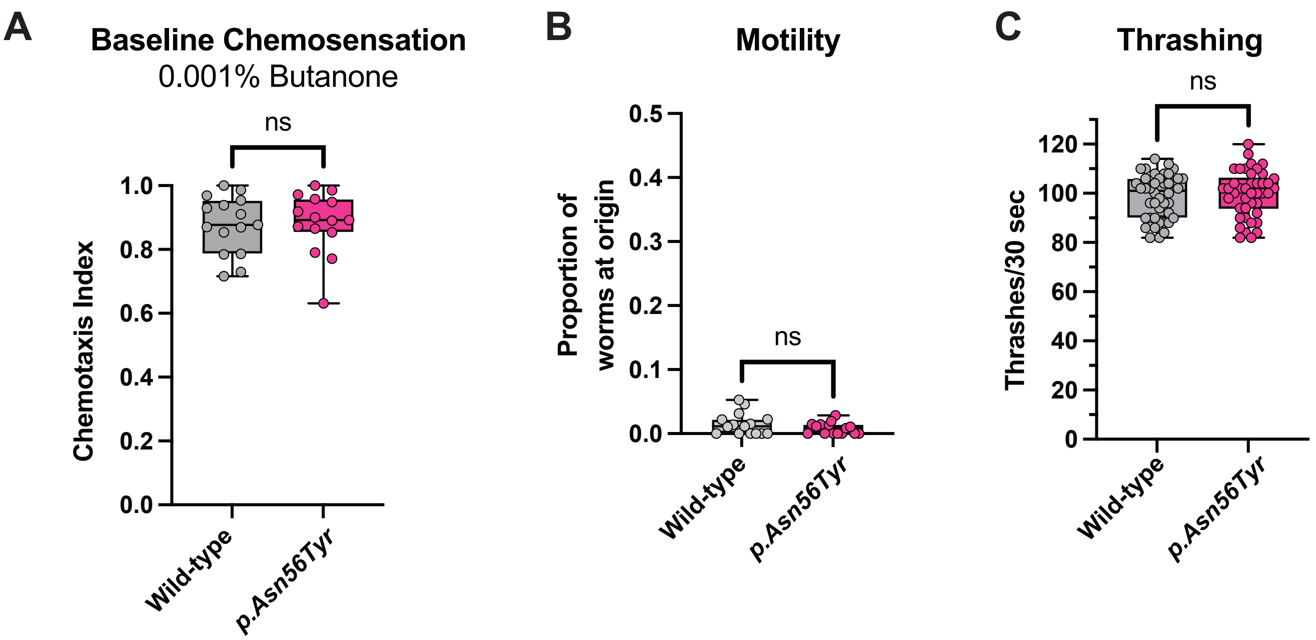

Supplemental Figure Legends:

Supplemental Figure 1. RNAi-based knockdown of RBPs does not have any detectable effect on learning ability. Box and whisker plots are shown for the learning timepoints from the STM/ITM assays for each RBP screened. Box and whisker plot: the center line denotes the median value (50th percentile) while the box contains the 25th to 75th percentiles. Whiskers mark the 5th and 95th percentiles. ns, not significant ($p>0.05$).

Supplemental Figure 2. Results of unbiased screen of 20 RBPs reveals both known and novel associative memory regulators. All RBPs with significantly altered memory are shown divided by memory timepoint. Box and whisker plot: the center line denotes the median value (50th percentile) while the box contains the 25th to 75th percentiles. Whiskers mark the 5th and 95th percentiles. $n \geq$ 5-10 per RNAi treatment. *$p<0.05$,**$p<0.01$, ***$p<0.001$, ****$p<0.0001$.

Supplemental Figure 3. Images generated from publicly available VISTA (Visualizing the Spatial Transcriptome of the *C. elegans* Nervous System) (1) reveal differences in expression across each of the *cey* RBPs at L4. While *cey-1* and *cey-4* have broad expression, *cey-2* is only located in eight neurons at L4.

Supplemental Figure 4. Data from multiple transcriptomic datasets as well as microscopy images reveal broad expression of CEY-1 in the nervous system of adult worms. (A) Neuron-specific and/or single-cell RNA-seq data compiled from five different publications suggest *cey-1* mRNA is expressed in the adult nervous system(2–7). (B) Diagram of microscopy images shown in (C). *C. elegans* head is labeled including the location of the pharynx and main neuronal ganglia/nerve rings. (C) A transcriptional *cey-1* reporter suggests the gene is broadly expressed in the neurons in the head at baseline conditions. Representative image of Day 2 adult worms with RFP-labeled neuronal nuclei (*unc-11(prom8)::2xNLS::TagRFP*)) and a nuclear GFPH2B *cey-1* promoter fusion *(cey1p::GFPH2B*) show colocalization.

Supplemental Figure 5. Knockdown of *cey-1* specifically in the adult nervous system does not impair butanone sensing, motility, or thrashing. (A) Naïve battery of both negative and positive odors reveals deficits in both nonanol and isoamyl alcohol chemotaxis, but not in benzaldehyde, pyrizine, or diacetyl chemotaxis at attractive concentrations. (B) Baseline chemosensation for butanone at neutral concentration of 10% is unaffected by neuron-specific knockdown of *cey-1*. n = 15 per RNAi treatment. (C) Baseline chemosensation for butanone at an attractive concentration of 0.001% is unaffected by neuron-specific knockdown of *cey-1*. n = 15 per RNAi treatment. (D) Thrashing is unaffected by neuron-specific loss of *cey-1*. $n \geq 30$ per RNAi treatment. (E) Motility, measured as % of worms at the origin of a chemotaxis plate, is unaffected by neuron-specific loss of *cey-1*. n = 15 per RNAi treatment. Box and whisker plot: the center line denotes the median value (50th percentile) while the box contains the 25th to 75th percentiles. Whiskers mark the 5th and 95th percentiles. **$p<0.01$, ****$p<0.0001$. ns, not significant ($p>0.05$).

Supplemental Figure 6. *cey-1* loss-of-function mutants (*cey-1*(*rrr12*)) as well as worms with neuron-specific rescue of *cey-1* ((*cey-1(rrr12)*; *rab-3p::CEY-1*)) have no deficits in butanone sensing or motility. (A) Baseline chemosensation for butanone at an attractive concentration of 0.001% is unaffected by whole-body loss of *cey-1*. n = 15 per genotype. (B) Motility, measured as % of worms at the origin of a chemotaxis plate, is unaffected by whole-body loss of *cey-1*. n = 15 per genotype. (C) Baseline chemosensation for butanone at an attractive concentration of 0.001% is unaffected by neuron-specific rescue of *cey-1*. n = 15 per genotype. (D) Motility, measured as % of worms at the origin of a chemotaxis plate, is unaffected by neuron-specific rescue of *cey-1*. n = 15 per genotype. Box and whisker plot: the center line denotes the median value (50th

percentile) while the box contains the 25th to 75th percentiles. Whiskers mark the 5th and 95th percentiles. ns, not significant (p>0.05).

Supplemental Figure 7. CNV gains and losses data for *YBX1* in DECIPHER(8). (A) Percentages of individuals reported with gain or loss CNVs that include *YBX1*. (B) Percentages of mechanisms of inheritance of gain and loss CNVs that include *YBX1*. (C) Size of CNV gain and losses that include *YBX1*. (D) Predictive scores for *YBX1* from gnomAD v.2.11(9) suggest that *YBX1* is intolerant to loss of function variants and is haploinsufficient. (E) Phenotypes of patients specifically with deletion/loss CNVs including *YBX1* include epicanthus, delayed speech and development, intellectual disability, and other neurological features. (F) Individuals reported in DECIPHER with CNV losses including *YBX1*, including the sex of the individual, the size of the deletion, the mechanism of inheritance (if known), the coordinates of the CNV loss, and the individual's reported phenotype.

Supplemental Figure 8. CNV gains and losses data for *YBX3* in DECIPHER(8). (A) Percentages of individuals reported with gain or loss CNVs that include *YBX3*. (B) Percentages of mechanisms of inheritance of gain and loss CNVs that include *YBX3*. (C) Size of CNV gain and losses that include *YBX3*. (D) Predictive scores for *YBX3* from gnomAD v.2.11(9) suggest that *YBX3* is tolerant to loss of function variants and is haplosufficient, suggesting variants may instead be deleterious by being dominant negative or gain-of-function. (E) Phenotypes of patients specifically with deletion/loss CNVs including *YBX3*, primarily intellectual disability, low-set ears, micrognathia, and other neurological features. (F) Individuals reported in DECIPHER with CNV losses including *YBX3*, including the sex of the individual, the size of the deletion, the mechanism of inheritance (if known), the coordinates of the CNV loss, and the individual's reported phenotype.

Supplemental Figure 9. Introduction of a point mutation from human individuals into cey-1 *(cey-1 (p.Asn56Tyr)* does not cause butanone sensing, motility, or thrashing deficits. (A) Baseline chemosensation for butanone at an attractive concentration of 0.001% is unaffected by the p.Asn56Tyr variant in *cey-1*. n = 15 per genotype. (E) Motility, measured as % of worms at the origin of a chemotaxis plate, is unaffected by p.Asn56Tyr variant in *cey-1*. n = 15 per genotype. (E) Thrashing is unaffected by the p.Asn56Tyr variant in *cey-1*. n $\geq$ 30 per RNAi treatment. Box and whisker plot: the center line denotes the median value (50th percentile) while the box contains the 25th to 75th percentiles. Whiskers mark the 5th and 95th percentiles. ns, not significant (p>0.05).

Supplemental References

1. Liska D, Wolfe Z, Norris A. VISTA: visualizing the spatial transcriptome of the C.elegans nervous system. Bioinformatics Advances. 2023 Sep 27;3(1):vbad127.
2. Kaletsky R, Yao V, Williams A, Runnels AM, Tadych A, Zhou S, et al. Transcriptome analysis of adult Caenorhabditis elegans cells reveals tissue-specific gene and isoform expression. PLoS Genet. 2018 Aug 10;14(8):e1007559.
3. Yao V, Kaletsky R, Keyes W, Mor DE, Wong AK, Sohrabi S, et al. An integrative tissue-network approach to identify and test human disease genes. Nat Biotechnol. 2018 Oct 22;
4. Arey R, Kaletsky R, Murphy CT. Profiling of presynaptic mRNAs reveals a role for axonal PUMILIOs in associative memory formation. BioRxiv. 2019 Aug 13;
5. Arey RN, Kaletsky R, Murphy CT. Nervous system-wide profiling of presynaptic mRNAs reveals regulators of associative memory. Sci Rep. 2019 Dec 30;9(1):20314.
6. Taylor SR, Santpere G, Weinreb A, Barrett A, Reilly MB, Xu C, et al. Molecular topography of an entire nervous system. Cell. 2021 Aug 5;184(16):4329–4347.e23.
7. Wang X, Jiang Q, Song Y, He Z, Zhang H, Song M, et al. Ageing induces tissue-specific transcriptomic changes in Caenorhabditis elegans. EMBO J. 2022 Apr 19;41(8):e109633.
8. Firth HV, Richards SM, Bevan AP, Clayton S, Corpas M, Rajan D, et al. DECIPHER: Database of Chromosomal Imbalance and Phenotype in Humans Using Ensembl Resources. Am J Hum Genet. 2009 Apr 1;84(4):524–33.
9. Chen S, Francioli LC, Goodrich JK, Collins RL, Kanai M, Wang Q, et al. A genome-wide mutational constraint map quantified from variation in 76,156 human genomes. BioRxiv. 2022 Mar 21;
